# The effects of high-intensity interval training on executive function in children and adolescents: a systematic review and meta-analysis

**DOI:** 10.3389/fpsyg.2026.1804529

**Published:** 2026-06-19

**Authors:** Hui Li, Shanan Yu, Meng Ye, Chen Wei, Min Huang

**Affiliations:** 1 School of Journalism and Communication, Shanghai University of Sport, Shanghai, China; 2 College of Sport, Shanghai University of Electric Power, Shanghai, China; 3 Rehabilitation Therapy Department, Bengbu Fourth People’s Hospital, Bengbu, China; 4 College of Sports Science, Shenyang Normal University, Shenyang, China; 5 School of Sports and Health, Shanghai Lixin University of Accounting and Finance, Shanghai, China

**Keywords:** adolescents, children, executive function, high-intensity interval training, meta-analysis

## Abstract

**Objective:**

To systematically evaluate the effects of high-intensity interval training (HIIT) on executive function in children and adolescents, and to quantify its effects on inhibitory control, working memory, and cognitive flexibility using a three-level meta-analysis.

**Methods:**

PubMed, Web of Science, The Cochrane Library, CNKI, Wanfang, and VIP databases were searched from inception to May 2026. Randomized controlled trials and non-randomized controlled intervention studies examining the effects of HIIT on executive function in children and adolescents were included. Two reviewers independently conducted study selection, data extraction, and risk-of-bias assessment. The risk of bias in randomized controlled trials was assessed using the Cochrane RoB 2 tool, while non-randomized intervention studies were assessed using the ROBINS-I tool. Hedges’ g was used as the effect size index. A three-level meta-analytic model was applied to account for the dependency of multiple effect sizes within the same study. Three-level Egger regression was used to assess publication bias and small-study effects, and sensitivity analyses were performed to examine the robustness of the findings.

**Results:**

A total of 11 studies were included, comprising 9 randomized controlled trials and 2 non-randomized controlled intervention studies, with 776 children and adolescents. The meta-analysis showed that HIIT significantly improved inhibitory control (Hedges’ g = 0.227, 95% CI: 0.066 to 0.388, *p* = 0.007), working memory (Hedges’ g = 0.368, 95% CI: 0.200 to 0.535, *p* < 0.001), and cognitive flexibility (Hedges’ g = 0.389, 95% CI: 0.184 to 0.593, *p* = 0.002). Sensitivity analyses showed that the positive effects of HIIT on inhibitory control and working memory remained statistically significant after excluding non-randomized studies. Subgroup analyses indicated positive effects across different work-to-rest ratios, intervention durations, and session durations; however, no significant between-subgroup differences were observed. Three-level Egger regression did not indicate significant funnel plot asymmetry or small-study effects. According to the GRADE assessment, the certainty of evidence was moderate for inhibitory control and working memory, but very low for cognitive flexibility.

**Conclusion:**

HIIT has positive effects on executive function in children and adolescents, particularly on inhibitory control and working memory, for which the certainty of evidence is moderate. Although HIIT also showed a positive effect on cognitive flexibility, this finding should be interpreted cautiously because of the limited number of studies and very low certainty of evidence. Further high-quality, large-sample studies with standardized intervention protocols and outcome measures are needed to confirm the effects of HIIT on executive function and to identify optimal training parameters for children and adolescents.

## 1 Introduction

Childhood and adolescence are critical periods not only for rapid physical growth and development but also for the maturation and refinement of cognitive and executive functions (Jin et al., 2022). Executive function plays an essential role in children’s and adolescents’ learning, daily life, and overall development. It refers to a set of higher-order cognitive processes that enable individuals to flexibly coordinate and regulate thoughts and behaviors in order to achieve goal-directed actions (Church et al., 2019; Ludyga et al., 2019). Executive function generally includes three core components: inhibitory control, working memory, and cognitive flexibility (Liang et al., 2022), all of which are important for healthy development in children and adolescents (Song et al., 2024). The development of executive function during childhood and adolescence has long-term implications for physical and mental health, academic achievement, and social success in adulthood (Wang, 2021). In contrast, deficits in executive function are associated not only with poorer learning ability but also with emotional problems, attention-deficit/hyperactivity disorder, and aggressive behaviors (Schoemaker et al., 2014). Increasing evidence suggests that physical exercise may promote the development of executive function in children and adolescents (Ludyga et al., 2016; Zhou and Jin, 2021; Northey et al., 2025). However, only approximately 20% of children and adolescents worldwide meet the World Health Organization recommendation of at least 60 min of moderate-to-vigorous physical activity per day (Liu et al., 2024). Therefore, time-efficient exercise strategies are needed to maximize the benefits of physical activity within limited available time.

High-intensity interval training (HIIT) is a form of exercise characterized by repeated short bouts of high-intensity activity, typically lasting 30 s to 4 min and performed at an intensity of ≥85% of maximal heart rate (HRmax), interspersed with periods of low-intensity exercise or passive recovery at approximately 40%–60% HRmax (Robinson et al., 2018; Zhang et al., 2019; Li, 2015). Compared with traditional continuous exercise, HIIT is time-efficient, effective, and relatively enjoyable, and has therefore been increasingly applied in studies aiming to improve physical fitness and cognitive function in children and adolescents. Previous studies have suggested that HIIT may have positive effects on various aspects of cognitive function in this population (Alves et al., 2021; Leahy et al., 2020), and it has been considered a feasible exercise approach for improving executive function in children and adolescents (Sun et al., 2024). However, the findings remain inconsistent. For example, one study reported that HIIT did not significantly improve inhibitory control in adolescents (Stenman et al., 2017). Existing systematic reviews and meta-analyses have provided preliminary evidence regarding the effects of HIIT on executive function in children and adolescents. Reyes-Amigo et al. (2022) included seven studies and found that HIIT had a positive effect on working memory but not on inhibitory control. Wang et al. (2023) further suggested that HIIT may improve inhibitory control and working memory in children, whereas the effects in adolescents remain limited.

Although previous studies have provided important evidence for understanding the relationship between HIIT and executive function in children and adolescents, further updates and methodological improvements are still needed. Previous meta-analyses have mainly used traditional two-level models, which may not adequately account for the dependence among multiple effect sizes when a single study reports several executive function outcomes or task indicators. Therefore, based on previous systematic reviews and meta-analyses, the present study included both randomized controlled trials and non-randomized controlled intervention studies, and applied a three-level meta-analytic model to account for the dependency of multiple effect sizes within the same study. In addition, the effects of HIIT were examined separately across the three core domains of executive function: inhibitory control, working memory, and cognitive flexibility. This study aims to provide evidence-based guidance for developing reasonable, efficient, and time-saving HIIT programs to promote executive function in children and adolescents.

## 2 Methods

This study was conducted in accordance with the Preferred Reporting Items for Systematic Reviews and Meta-Analyses (PRISMA) guidelines (Page et al., 2021). The review protocol was registered in the International Prospective Register of Systematic Reviews (PROSPERO; registration number: CRD42024609179).

### 2.1 Search strategy

Two reviewers independently searched six databases, including PubMed, Web of Science, The Cochrane Library, Wanfang, VIP, and China National Knowledge Infrastructure (CNKI), to identify randomized controlled trials and non-randomized controlled intervention studies examining the effects of HIIT on executive function in children and adolescents. The search covered all records from database inception to May 2026. In addition, the reference lists of the included studies were manually searched to identify potentially eligible studies. The detailed search strategy is presented in Supplementary Table S1.

### 2.2 Inclusion and exclusion criteria

#### 2.2.1 Inclusion criteria

Studies were included if they met the following criteria:

(1) Participants were healthy children or adolescents aged 6–18 years.
(2) The intervention group received HIIT.
(3) The outcome measures included executive function.
(4) The study design was a randomized controlled trial (RCT) or a non-randomized controlled trial/intervention study (NRCT).

#### 2.2.2 Exclusion criteria

Studies were excluded if they met any of the following criteria:

(1) Participants were special populations, such as children with autism spectrum disorder, attention-deficit/hyperactivity disorder, obesity, or other specific conditions.
(2) Data were incomplete or could not be extracted.
(3) The publication was a review article, conference abstract, or conference paper.

### 2.3 Study selection and data extraction

All retrieved records were imported into EndNote for duplicate removal. Two reviewers independently screened the studies according to the inclusion and exclusion criteria, extracted relevant data, and cross-checked the extracted information. Any disagreement was resolved through discussion; if consensus could not be reached, a third reviewer was consulted to make the final decision. The extracted information included the first author, publication year, study design, country, participants’ age, sample size of each group, HIIT intervention characteristics, and type of executive function outcome.

### 2.4 Quality assessment

The risk of bias was assessed using appropriate tools according to the design of the included studies. For randomized controlled trials, the revised Cochrane risk-of-bias tool for randomized trials (RoB 2) was used (Yin et al., 2025). For non-randomized controlled trials or quasi-experimental studies, the Risk Of Bias In Non-randomized Studies of Interventions tool (ROBINS-I) was used (Sterne et al., 2016).

The RoB 2 tool assesses five domains of bias: bias arising from the randomization process, bias due to deviations from intended interventions, bias due to missing outcome data, bias in measurement of the outcome, and bias in selection of the reported result. Each domain was judged as “low risk of bias,” “some concerns,” or “high risk of bias,” and an overall risk-of-bias judgment was then made.

The ROBINS-I tool is used to assess the risk of bias in non-randomized intervention studies across seven domains: bias due to confounding, bias in selection of participants into the study, bias in classification of interventions, bias due to deviations from intended interventions, bias due to missing data, bias in measurement of outcomes, and bias in selection of the reported result. Each domain was judged as “low,” “moderate,” “serious,” or “critical” risk of bias, and an overall risk-of-bias judgment was then made.

All included studies were independently assessed by two reviewers. Disagreements were resolved through discussion; if consensus could not be reached, a third reviewer was consulted.

### 2.5 Statistical analysis

All statistical analyses were performed using R software. To account for the dependency among multiple effect sizes within the same study, a three-level random-effects meta-analysis model was used (Liu et al., 2026). Standardized mean differences and their variances were calculated based on post-intervention means, standard deviations, and sample sizes of the intervention and control groups. Hedges’ g and its 95% confidence interval (CI) were used as the effect size measure. Effect sizes were interpreted as follows: *g* ≤ 0.20 was considered a small effect, 0.20–0.49 a small-to-moderate effect, 0.50–0.79 a moderate effect, and *g* ≥ 0.80 a large effect. Publication bias was assessed using funnel plots and Egger’s regression test.

When the direction of outcome measures was inconsistent across studies, effect sizes were harmonized according to the recommendations of the *Cochrane Handbook for Systematic Reviews of Interventions*. For outcomes in which lower values indicated better performance, such as reaction time, the mean values were multiplied by −1 before calculating the effect size (Chandler et al., 2019). Thus, all effect sizes were coded so that positive values indicated better executive function performance in the HIIT group than in the control group.

### 2.6 Publication bias and sensitivity analysis

Because some studies reported multiple effect sizes, these effect sizes may not have been statistically independent. Therefore, a three-level Egger regression model was used to assess publication bias and small-study effects. In this model, the effect size was treated as the dependent variable, the standard error of the effect size was included as a moderator, and random effects were specified at both the study level and the within-study effect-size level. A statistically significant regression coefficient for the standard error was considered to indicate possible funnel plot asymmetry or small-study effects.

To examine the robustness of the findings, sensitivity analyses were further performed. For studies contributing multiple effect sizes, leave-one-study-out sensitivity analyses were conducted at the study level.

## 3 Results

### 3.1 Literature search results

**Figure 1:**
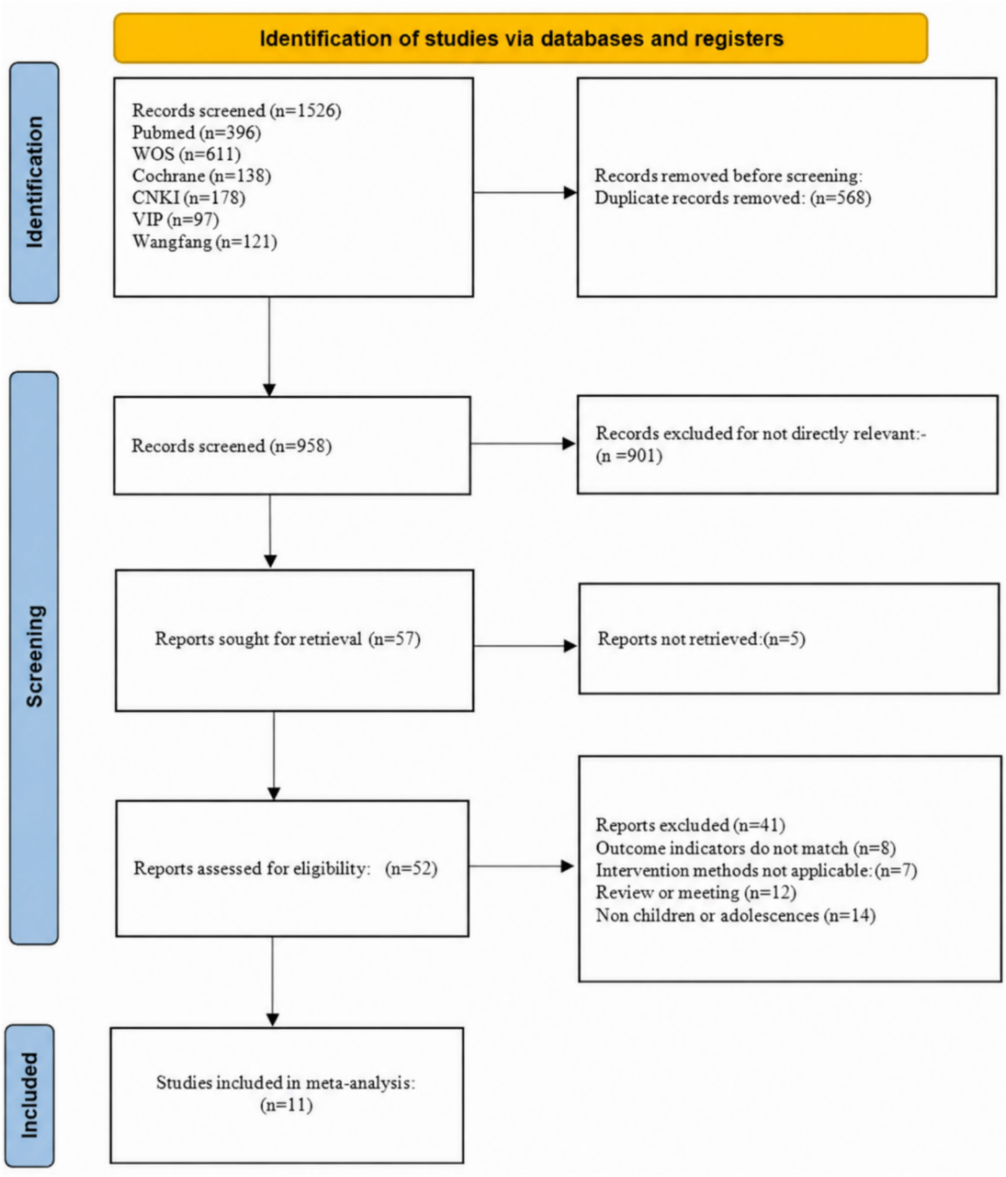
Flowchart of literature screening.

A total of 1,526 records were retrieved. After removing 568 duplicate records, 901 irrelevant records were excluded during the initial screening. Five reports could not be retrieved, and 11 studies were finally included. The literature screening process is shown in Figure 1.

### 3.2 Risk-of-bias assessment

A total of 11 studies were included (Wang, 2021; Cai, 2020; Zhang, 2019; Zhang, 2020; Ben-Zeev et al., 2020; Costigan et al., 2016; Leahy et al., 2020; Moreau et al., 2017; Williams et al., 2022; Liu et al., 2025; Tottori et al., 2019), including 9 RCTs (Wang, 2021; Cai, 2020; Zhang, 2019; Zhang, 2020; Costigan et al., 2016; Leahy et al., 2020; Moreau et al., 2017; Williams et al., 2022; Liu et al., 2025) and 2 NRCTs (Ben-Zeev et al., 2020; Tottori et al., 2019). The RCTs were assessed using the RoB 2 tool. The RoB 2 assessment showed that 1 study was rated as having a low risk of bias, 7 studies were rated as having some concerns, and 1 study was rated as having a high risk of bias. The main sources of bias were insufficient reporting of the randomization process, difficulty in blinding participants and intervention personnel during the intervention, and selective reporting in some studies.

**Figure 2:**
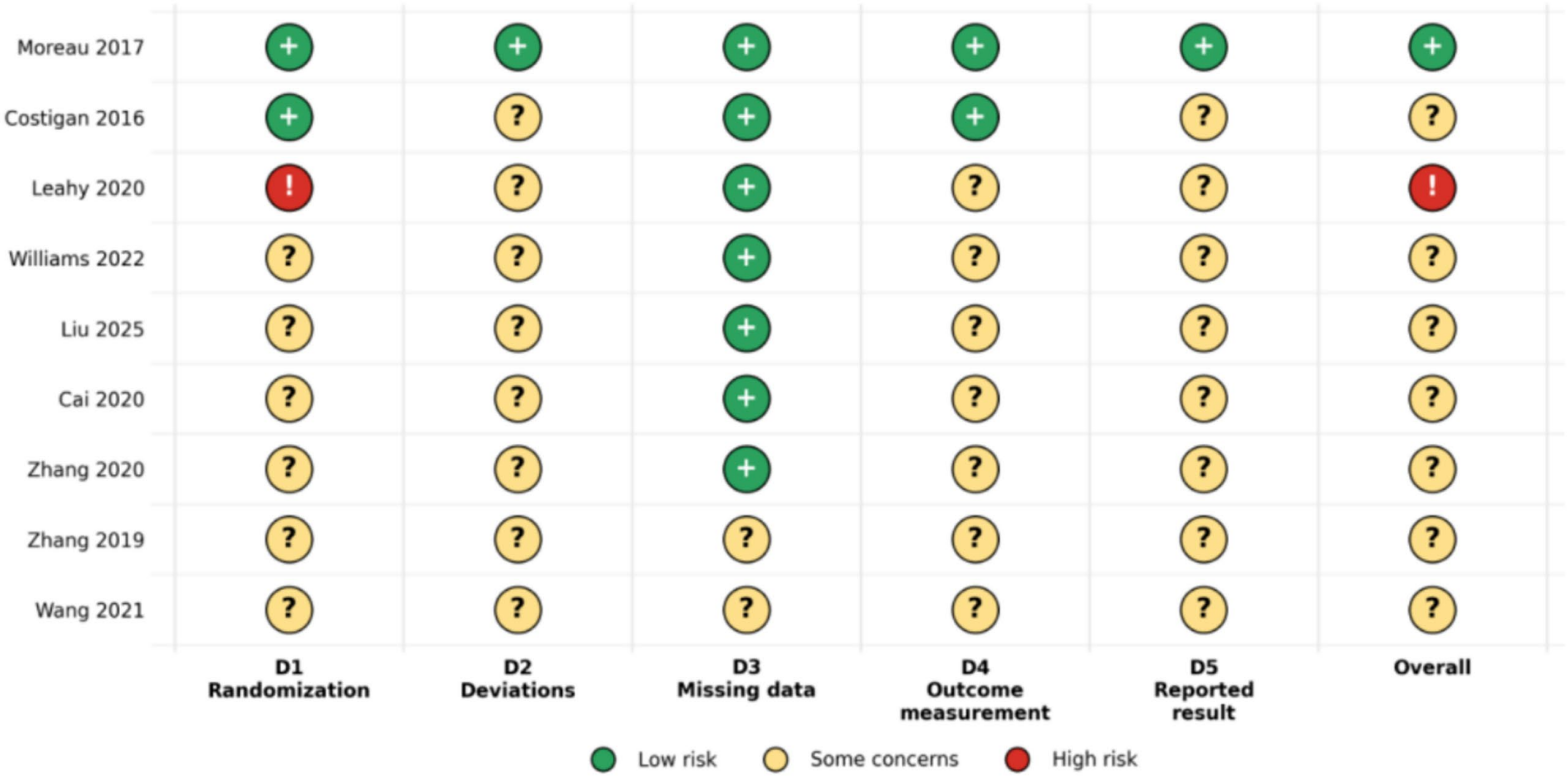
RoB 2 risk-of-bias assessment.

**Figure 3:**
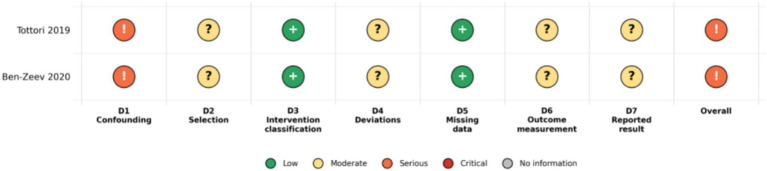
ROBINS-I risk-of-bias assessment.

The ROBINS-I assessment showed that the 2 NRCTs were both rated as having a serious overall risk of bias. The main source of bias was confounding, as neither study used individual randomization. The classification of interventions was relatively clear, and the risk of bias due to missing outcome data was low. However, because outcome assessors were not blinded, bias in outcome measurement and bias in selection of the reported result were both rated as moderate risk (see Figures 2, 3).

### 3.3 Characteristics of included studies

A total of 11 studies were included, involving 776 participants, with 400 participants in the intervention groups and 376 participants in the control groups. Participants in the intervention groups received HIIT, whereas those in the control groups received moderate-intensity continuous training (MICT), regular physical education classes, or usual physical activity. The age of participants in the included studies ranged from 8 to 16 years. The HIIT interventions lasted 2 to 12 weeks, with a frequency of 3 to 5 sessions per week and a duration of 6 to 30 min per session. The basic characteristics of the included studies are presented in Table 1.

**Table 1:** Basic characteristics of the included studies.

Reference	Country	Study type	*n* (T/C)	Age (T/C)/Years	Intervention (T/C)	Exercise protocol	WRR	Assessment tools
Wang (2021)	China	RCT	32/32	11.68 ± 0.28/11.61 ± 0.27	T: HIIT C: Regular Physical Education Class	F: 3 times/wk. T: 20 min D: 8 weeks	1.1 ~ 1.2	Flanker, N-back, More-odd shifting
Cai (2020)	China	RCT	30/30	8 ~ 9	T: HIIT C: Regular Physical Education Class	F: 3 times/wk. T: 10 min D: 8 weeks	1:3	Flanker, N-back, More-odd shifting
Zhang (2019)	China	RCT	23/22	10.37 ± 0.49/10.31 ± 0.48	T: HIIT C: Continuous Aerobic Training	F: 3 times/wk. T: 15 min D: 6 weeks	1:1	Stroop
Zhang (2020)	China	RCT	16/16	12.77 ± 0.44/12.67 ± 0.49	T: HIIT C: Regular Physical Education Class	F: 3 times/wk. T: 30 min D: 8 weeks	1:2	Stroop
Ben-Zeev et al. (2020)	Israel	NRCT	20/20	12 ~ 13	T: HIIT C: Regular Physical Education Class	F: 3 times/wk. T: 20 min D: 12 weeks	1:1	Stroop
Costigan et al. (2016)	Australia	RCT	T1:21/ T2: 22/C:22	T1:15.7 ± 0.7/ T2: 15.5 ± 0.6/C:15.6 ± 0.6	T1: Aerobic HIIT T2: Resistance HIIT C: Regular Physical Activity	F: 3 times/wk. T: 8–10 min D: 8 weeks	1:1	The trail making test
Leahy et al. (2020)	Australia	RCT	33/29	16.2 ± 0.4/16.2 ± 0.4	T: HIIT C: Regular Physical Education Class	F: 3 times/wk. T: 12–20 min D: 14 weeks	1:1	Flanker, N-back
Moreau et al. (2017)	New Zealand	RCT	152/153	9.96 ± 1.68/9.87 ± 1.81	T: HIIT C: Video Game Workouts	F: 5 times/wk. T: 6 min D: 6 weeks	1:1 ~ 1:2.5	Stroop, Flanker, Go/Nogo, N-back, Backward digit span, Backward Corsi blocks
Williams et al. (2022)	United Kingdom	RCT	8/8	11.8 ± 0.2/11.6 ± 0.4	T: HIIT C: Regular Physical Activity	F: 3 times/wk. T: 6–8 min D: 2 weeks	1:5	Stroop, Flanker, Sternberg
Liu et al. (2025)	China	RCT	18/19	9–10	T: HIIT C: Regular Physical Activity	F: 5 times/wk. T: 10 min D: 8 weeks	1:1	Stroop
Tottori et al. (2019)	Japan	NRCT	29/27	10.0 ± 1.0/10.4 ± 1.1	T: HIIT C: Regular Physical Activity	F: 3 times/wk. T: 8–10 min D: 4 weeks	1:1	Digit forward span, Digit backward test

T, experimental group; C, control group; RCT, randomized controlled trial; NRCT, non-randomized controlled trial; WRR, work-to-rest ratio; F, frequency; T, Time; D, Duration.

### 3.4 Meta-analysis

**Figure 4:**
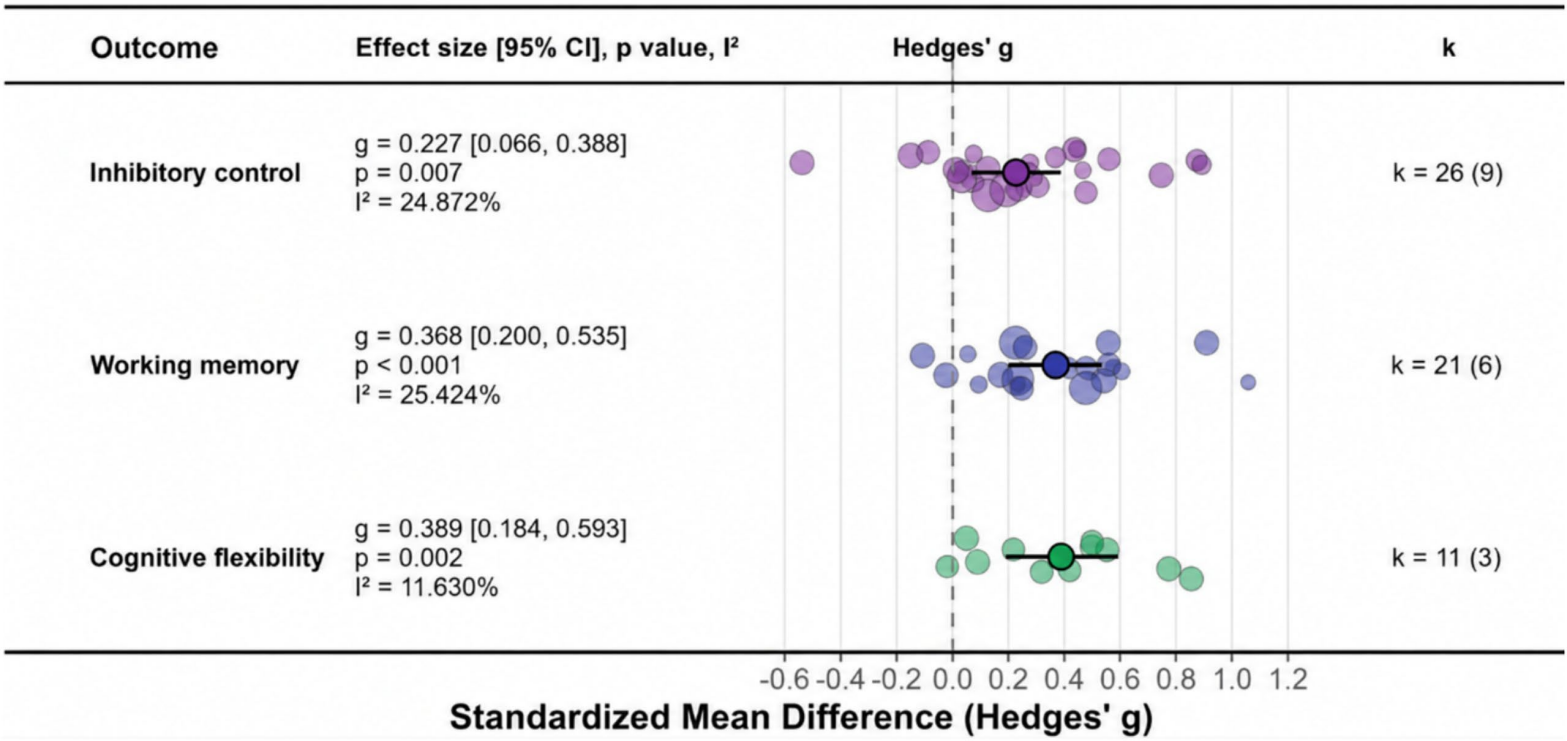
Orchard plot of the effects of HIIT on executive function in children and adolescents.

The meta-analysis of the effects of HIIT on executive function in children and adolescents is shown in Figure 4. A total of 9 studies (Wang, 2021; Cai, 2020; Zhang, 2019; Zhang, 2020; Ben-Zeev et al., 2020; Leahy et al., 2020; Moreau et al., 2017; Williams et al., 2022; Liu et al., 2025) with 26 effect sizes examined the effect of HIIT on inhibitory control. The results showed that HIIT significantly improved inhibitory control in children and adolescents (Hedges’ g = 0.227, 95% CI: 0.066 to 0.388, *p* = 0.007). A total of 6 studies (Wang, 2021; Cai, 2020; Leahy et al., 2020; Moreau et al., 2017; Williams et al., 2022; Tottori et al., 2019) with 21 effect sizes examined the effect of HIIT on working memory. The results showed that HIIT significantly improved working memory in children and adolescents (Hedges’ g = 0.368, 95% CI: 0.200 to 0.535, *p* < 0.001). A total of 3 studies (Wang, 2021; Cai, 2020; Costigan et al., 2016) with 11 effect sizes examined the effect of HIIT on cognitive flexibility. The results showed that HIIT significantly improved cognitive flexibility in children and adolescents (Hedges’ g = 0.389, 95% CI: 0.184 to 0.593, *p* = 0.002).

### 3.5 Sensitivity analysis

To examine the influence of NRCTs on the pooled results, sensitivity analyses were further performed. For inhibitory control, the main analysis including both RCTs and NRCTs showed a significant positive effect of HIIT on inhibitory control in children and adolescents (Hedges’ g = 0.227, 95% CI: 0.066 to 0.388, *p* = 0.007). After excluding NRCTs and retaining only RCTs, the pooled effect size decreased slightly but remained statistically significant (Hedges’ g = 0.187, 95% CI: 0.053 to 0.317, *p* = 0.006). For working memory, the main analysis also showed a significant positive effect of HIIT (Hedges’ g = 0.368, 95% CI: 0.200 to 0.535, *p* < 0.001). After excluding NRCTs, the pooled effect remained statistically significant (Hedges’ g = 0.385, 95% CI: 0.180 to 0.590, p < 0.001). These findings suggest that the main results of the present study were relatively robust.

### 3.6 Subgroup analysis

To further explore potential sources of heterogeneity in the effects of HIIT on executive function in children and adolescents, subgroup analyses were conducted for inhibitory control and working memory, the two domains with significant pooled effects. The subgroup cut-offs were determined based on common HIIT intervention characteristics, practical feasibility in youth exercise settings, and the distribution of the included studies. Subgroups were defined by intervention duration, session duration, and work-to-rest ratio (WRR). An 8-week cut-off was used to distinguish shorter from longer interventions, a 20-min cut-off was used to distinguish shorter school-feasible HIIT sessions from longer sessions, and a WRR cut-off of 1 was used to distinguish whether the high-intensity work period was shorter than the recovery period. For inhibitory control, subgroup analyses showed significant positive effects in the WRR < 1, intervention duration ≥8 weeks, and session duration <20 min subgroups. In contrast, the WRR ≥ 1, intervention duration <8 weeks, and session duration ≥20 min subgroups did not reach statistical significance. However, no significant between-subgroup differences were observed for WRR, intervention duration, or session duration (all *p* > 0.05). For working memory, significant positive effects were observed across different WRR, intervention duration, and session duration subgroups, suggesting that the beneficial effect of HIIT on working memory in children and adolescents may be relatively consistent across different intervention protocols. However, between-subgroup differences were not statistically significant (all *p* > 0.05), as shown in Table 2.

**Table 2:** Subgroup analysis of the effects of HIIT on executive function in children and adolescents.

Outcome	Subgroup		*k*	Hedges’ g (95% CI)	P-within	P-between
Inhibitory control	WRR	≥1	11	0.171(−0.079, 0.420)	0.172	0.458
		<1	15	0.299 (0.051, 0.547)	0.020	
	Duration	≥8	14	0.290 (0.063, 0.517)	0.014	0.434
		<8	12	0.150 (−0.135, 0.435)	0.288	
	Time	≥20	6	0.306 (−0.057, 0.670)	0.094	0.662
		<20	20	0.216 (0.002, 0.430)	0.048	
Working memory	WRR	≥1	10	0.305 (0.039, 0.570)	0.026	0.449
		<1	11	0.442 (0.179, 0.705)	0.002	
	Duration	≥8	8	0.415 (0.123, 0.707)	0.007	0.715
		<8	13	0.345 (0.081, 0.610)	0.013	
	Time	≥20	2	0.549 (0.064, 1.035)	0.029	0.415
		<20	19	0.343 (0.163, 0.522)	<0.001	

### 3.7 Publication bias

**Figure 5:**
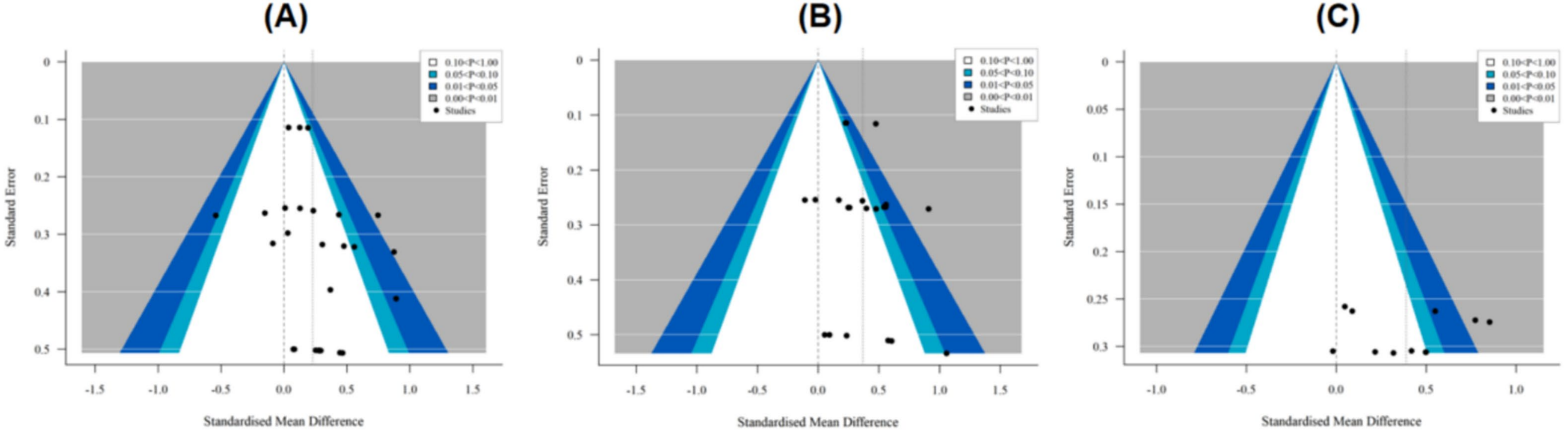
Funnel plots for publication bias assessment. **(A)** Inhibitory control; **(B)** working memory; **(C)** cognitive flexibility.

The results of the three-level Egger regression test showed that the regression coefficients of the standard error of the effect size were not statistically significant for inhibitory control, working memory, or cognitive flexibility, with *p* values of 0.228, 0.584, and 0.802, respectively. These results suggest that no clear evidence of funnel plot asymmetry or small-study effects was detected. However, because only three studies were included for the cognitive flexibility outcome, the statistical power of the publication bias test was limited, and the corresponding result should therefore be interpreted with caution (see Figure 5).

### 3.8 Certainty of evidence

The GRADE assessment showed that the certainty of evidence for the effects of HIIT on inhibitory control and working memory in children and adolescents was moderate. Both outcomes were downgraded by one level because of serious risk of bias in the included studies, as shown in Table 3. The certainty of evidence for cognitive flexibility was rated as very low. The main reasons were serious risk of bias in the included studies, imprecision due to the small number of studies and limited statistical power, and potential publication bias related to the high proportion of small-sample studies.

**Table 3:** GRADE quality assessment of evidence.

Outcome	*k*	Risk of bias	Inconsistency	Indirectness	Imprecision	Publication bias	Quality of evidence
Inhibitory control	9 (26)	Serious	Not serious	Not serious	Not serious	Not serious	Moderate
Working memory	6 (21)	Serious	Not serious	Not serious	Not serious	Not serious	Moderate
Cognitive flexibility	3 (11)	Serious	Not serious	Not serious	Serious	Serious	Very low

## 4 Discussion

This study systematically evaluated the effects of HIIT on executive function in children and adolescents using a systematic review and three-level meta-analysis. The GRADE assessment showed that the certainty of evidence was moderate for the effects of HIIT on inhibitory control and working memory, but very low for cognitive flexibility. The main reasons for downgrading the certainty of evidence included risk of bias in some included studies, the small number of studies related to cognitive flexibility, instability of the effect estimates, and potential publication bias. Therefore, more rigorously designed randomized controlled trials with larger sample sizes and more consistent outcome measures are needed to further verify these findings.

The results of this study showed that HIIT had a significant positive effect on executive function in children and adolescents, as reflected by improvements in inhibitory control, working memory, and cognitive flexibility. These findings are generally consistent with previous studies (Hu et al., 2021). The potential mechanisms by which HIIT improves executive function may be related to its positive regulation of the central nervous system and metabolic function. Previous studies have shown that HIIT may promote the secretion of brain-derived neurotrophic factor, improve oxidative stress and mitochondrial function, and increase brain lactate levels and utilization, thereby promoting neuroplasticity and cognitive function (Liu et al., 2024; De Miguel et al., 2021; Miller et al., 2019). Lactate is not only a metabolic product during exercise but may also act as an important energy substrate and signaling molecule involved in the regulation of brain function. A moderate increase in lactate levels may provide additional energy support for the brain and activate neuroplasticity-related pathways, thereby contributing to improved executive function. However, previous studies have suggested that during high-intensity exercise, substantial metabolic resources may be required to maintain exercise performance and motor control, potentially reducing the resources available for cognitive processing and thereby negatively affecting executive function (Dietrich, 2003; Kao et al., 2023).

From the perspective of different domains of executive function, this study found that HIIT significantly improved inhibitory control (Hedges’ g = 0.227), working memory (Hedges’ g = 0.368), and cognitive flexibility (Hedges’ g = 0.389), although the magnitude of the effects differed across domains. This may be related to differences in the neural basis and task characteristics of different executive function components. Inhibitory control is mainly associated with the prefrontal cortex, anterior cingulate cortex, and conflict-monitoring networks; working memory relies more on the dorsolateral prefrontal cortex and frontoparietal networks; and cognitive flexibility is closely related to the prefrontal cortex, parietal cortex, and task-switching networks (Zheng et al., 2014). Physiological adaptations induced by HIIT may affect different brain regions and cognitive networks to varying degrees, which may partly explain the differences in effect sizes across executive function domains. In addition, the behavioral tasks used in the included studies varied, such as the Stroop task, Flanker task, n-back task, and Trail Making Test. Although these tasks are commonly used to assess executive function, they differ in cognitive load and measurement focus, which may also influence the pooled effect estimates for different executive function domains.

Appropriate HIIT interventions may positively influence executive function in children and adolescents by improving cardiorespiratory fitness, enhancing cerebral blood flow and oxygenation, promoting metabolic adaptation, and increasing neuroplasticity. When the WRR is less than 1, the recovery period is longer than the high-intensity exercise period, which may help children and adolescents maintain better physiological and psychological states during exercise, avoid excessive fatigue, and provide relatively sufficient oxygen and glucose supply to the brain (Beinlich et al., 2024). In addition, longer HIIT interventions may be more likely to induce stable cardiorespiratory and neural adaptations, which may explain the significant effects observed in the subgroup with an intervention duration of ≥8 weeks. Furthermore, excessively long sessions or an inappropriate work-to-rest ratio may increase subjective fatigue, psychological stress, and negative emotional experiences in children and adolescents, thereby affecting cognitive performance. According to the strength model of self-control, individuals need to continuously mobilize self-control resources during high-intensity exercise to maintain exercise intensity and movement execution. If the exercise load is too high or recovery is insufficient, self-control resources may be depleted, which may negatively affect subsequent executive function performance (Liu et al., 2024; Boat and Cooper, 2019). Previous studies have also suggested that HIIT may induce stronger negative affective responses than moderate-intensity continuous training (Dong et al., 2022; Hu et al., 2021; Saanijoki et al., 2015). Therefore, when designing HIIT programs for children and adolescents, their age characteristics, physiological development, exercise experience, and psychological acceptance should be fully considered to avoid the adverse effects of excessive intensity or overly long exercise duration.

Although the pooled effect for cognitive flexibility reached statistical significance, this outcome was based on only 3 studies, and no formal subgroup analysis was therefore conducted. Cognitive flexibility may be influenced by multiple factors, including age, task complexity, learning experience, and neurodevelopmental level. Children and adolescents are in a critical period of executive function development, and the developmental trajectories of inhibitory control, working memory, and cognitive flexibility may differ across age stages. Previous meta-analytic evidence has suggested that age may moderate the effects of exercise on executive function (Ludyga et al., 2016). The age criterion of 6–18 years was predefined because this range broadly covers school-aged children and adolescents, which was the target population of the present review. However, the actual age range of participants in the included studies was 8–16 years. Given the substantial neurocognitive changes that occur during this developmental period, age-related heterogeneity should be considered when interpreting the findings. Due to the limited number of included studies, further subgroup analyses by age stage could not be performed. Therefore, future studies should distinguish between different developmental stages in children and adolescents and examine the age-specific effects of HIIT on different domains of executive function.

## 5 Limitations

This study has several limitations. First, some included studies used MICT as the control condition rather than a completely inactive control group, which may have reduced the between-group differences between HIIT and the control condition and thus influenced the pooled effect size. Second, although this study classified outcome measures into inhibitory control, working memory, and cognitive flexibility, the behavioral task paradigms used across studies differed in terms of task difficulty, cognitive load, and scoring methods, which may have increased the imprecision of the results. Third, although no significant publication bias was detected (*p* > 0.05), the limited number of included studies reduced the statistical power of the test, and the possibility of publication bias cannot be completely excluded. Fourth, this study mainly assessed changes in executive function using behavioral outcomes, while neuroimaging, electroencephalographic, or blood biomarker indicators were rarely reported. Therefore, the neurophysiological mechanisms underlying the effects of HIIT on executive function could not be further explained. Future studies should use more standardized executive function assessment tasks and incorporate neuroimaging, EEG activity, BDNF, lactate, cardiorespiratory fitness, and other indicators to further clarify the potential mechanisms by which HIIT affects executive function in children and adolescents.

## 6 Conclusion

The findings suggest that HIIT has a positive effect on executive function in children and adolescents, as reflected by improvements in inhibitory control, working memory, and cognitive flexibility. However, because only a small number of studies examined cognitive flexibility and the certainty of evidence for this outcome was very low, this finding should be interpreted with caution. Subgroup analyses showed positive effects across different WRR, intervention duration, and session duration subgroups, but no significant between-subgroup differences were observed. Therefore, current evidence does not confirm that specific intervention parameters significantly moderate the effects of HIIT on executive function. Future high-quality studies with larger sample sizes, standardized HIIT protocols, and more uniform executive function testing paradigms are needed to improve the comparability of findings and the reliability of pooled effect estimates.

## Data Availability

The datasets used and/or analyzed during the current study are available from the corresponding author upon reasonable request. Requests to access these datasets should be directed to huangmin_slu@163.com.
