# Supplementary material for: The effects of high-intensity interval training on executive function in children and adolescents: a systematic review and meta-analysis
Source: Front Psychol. 2026 Jun 19;17:1804529. doi: 10.3389/fpsyg.2026.1804529 (PMC13329722; doi:10.3389/fpsyg.2026.1804529)
Supplement: Supplementary file 1 [file Table_1.docx]

Table 1. Literature search strategy

| Database | Search steps |
| --- | --- |
| **PubMed and The Cochrane Library** | **#1** ("high-intensity interval training"[Title/Abstract] OR "high-intensity intermittent training"[Title/Abstract] OR "high-intensity interval exercise"[Title/Abstract] OR "high-intensity functional training"[Title/Abstract] OR "sprint interval training"[Title/Abstract] OR "HIIT"[Title/Abstract] OR "HIIE"[Title/Abstract] OR "HIT"[Title/Abstract] OR "HIFT"[Title/Abstract]) **#2** ("executive function"[Title/Abstract] OR "executive functions"[Title/Abstract] OR "executive control"[Title/Abstract] OR "cognitive control"[Title/Abstract] OR "attention"[Title/Abstract] OR "attentional control"[Title/Abstract] OR "working memory"[Title/Abstract] OR "updating"[Title/Abstract] OR "inhibition"[Title/Abstract] OR "inhibitory control"[Title/Abstract] OR "response inhibition"[Title/Abstract] OR "cognitive flexibility"[Title/Abstract] OR "shifting"[Title/Abstract] OR "task switching"[Title/Abstract] OR "Stroop"[Title/Abstract] OR "Flanker"[Title/Abstract] OR "Go/No-go"[Title/Abstract] OR "Go No-Go"[Title/Abstract] OR "n-back"[Title/Abstract] OR "Trail Making Test"[Title/Abstract] OR "TMT"[Title/Abstract] OR "Digit Span"[Title/Abstract] OR "Corsi"[Title/Abstract] OR "Sternberg"[Title/Abstract]) **#3** ("child"[Title/Abstract] OR "children"[Title/Abstract] OR "childhood"[Title/Abstract] OR "pediatric"[Title/Abstract] OR "paediatric"[Title/Abstract] OR "pupil"[Title/Abstract] OR "student"[Title/Abstract] OR "school-aged"[Title/Abstract] OR "youth"[Title/Abstract] OR "adolescent"[Title/Abstract] OR "adolescents"[Title/Abstract] OR "teenager"[Title/Abstract] OR "teenagers"[Title/Abstract]) **#4** ("randomized controlled trial"[Publication Type] OR "controlled clinical trial"[Publication Type] OR random*[Title/Abstract] OR trial[Title/Abstract] OR intervention[Title/Abstract] OR "controlled trial"[Title/Abstract] OR "quasi-experimental"[Title/Abstract] OR "non-randomized"[Title/Abstract] OR "nonrandomized"[Title/Abstract]) **#5** #1 AND #2 AND #3 AND #4 |
| **Web of Science** | **#1** TS=("high-intensity interval training" OR "high-intensity intermittent training" OR "high-intensity interval exercise" OR "high-intensity functional training" OR "sprint interval training" OR "HIIT" OR "HIIE" OR "HIT" OR "HIFT") **#2** TS=("executive function" OR "executive functions" OR "executive control" OR "cognitive control" OR "attention" OR "attentional control" OR "working memory" OR "updating" OR "inhibition" OR "inhibitory control" OR "response inhibition" OR "cognitive flexibility" OR "shifting" OR "task switching" OR "Stroop" OR "Flanker" OR "Go/No-go" OR "Go No-Go" OR "n-back" OR "Trail Making Test" OR "TMT" OR "Digit Span" OR "Corsi" OR "Sternberg") **#3** TS=("child" OR "children" OR "childhood" OR "pediatric" OR "paediatric" OR "pupil" OR "student" OR "school-aged" OR "youth" OR "adolescent" OR "adolescents" OR "teenager" OR "teenagers") **#4** TS=("randomized controlled trial" OR "randomised controlled trial" OR "controlled clinical trial" OR random* OR trial OR intervention OR "controlled trial" OR "quasi-experimental" OR "non-randomized" OR "nonrandomized") **#5** #1 AND #2 AND #3 AND #4 |
| **CNKI** | 主题 =（高强度间歇运动 OR 高强度间歇训练 OR 高强度间歇性训练 OR 高强度功能性训练 OR 冲刺间歇训练 OR 冲刺训练 OR HIIT OR HIIE OR HIT OR HIFT）AND 主题 =（执行功能 OR 执行控制 OR 认知控制 OR 注意 OR 注意控制 OR 抑制控制 OR 抑制功能 OR 反应抑制 OR 工作记忆 OR 刷新 OR 认知灵活性 OR 转换 OR 任务转换 OR Stroop OR Flanker OR Go/No-go OR n-back OR Trail Making Test OR TMT OR 数字广度 OR Corsi OR Sternberg）AND 主题 =（儿童 OR 少儿 OR 学生 OR 小学生 OR 中学生 OR 青少年 OR 青春期）AND 主题 =（随机 OR 对照 OR 干预 OR 试验 OR 准实验 OR 非随机） |
| **Wanfang and VIP** | 主题 =（高强度间歇运动 OR 高强度间歇训练 OR 高强度间歇性训练 OR 高强度功能性训练 OR 冲刺间歇训练 OR 冲刺训练 OR HIIT OR HIIE OR HIT OR HIFT）AND 主题 =（执行功能 OR 执行控制 OR 认知控制 OR 注意 OR 注意控制 OR 抑制控制 OR 抑制功能 OR 反应抑制 OR 工作记忆 OR 刷新 OR 认知灵活性 OR 转换 OR 任务转换 OR Stroop OR Flanker OR Go/No-go OR n-back OR Trail Making Test OR TMT OR 数字广度 OR Corsi OR Sternberg）AND 主题 =（儿童 OR 少儿 OR 学生 OR 小学生 OR 中学生 OR 青少年 OR 青春期）AND 主题 =（随机 OR 对照 OR 干预 OR 试验 OR 准实验 OR 非随机） |
